# Otitis media with effusion in children younger than 1 year

**DOI:** 10.1016/j.rppede.2016.01.003

**Published:** 2016

**Authors:** Renata Cantisani Di Francesco, Vivian Boschesi Barros, Rafael Ramos

**Affiliations:** a Faculdade de Medicina, Universidade de São Paulo (USP), São Paulo, SP, Brazil; b Hospital das Clínicas, Faculdade de Medicina, Universidade de São Paulo (USP), São Paulo, SP, Brazil

**Keywords:** Otitis media with effusion, Infant, Risk factors

## Abstract

**Objective:**

To determine the prevalence of otitis media with effusion in children younger than 1 year and its association with the season of the year, artificial feeding, environmental and perinatal factors.

**Methods:**

Retrospective study of 184 randomly included medical records from a total of 982 healthy infants evaluated for hearing screening tests. Diagnosis of otitis media with effusion was based on otoscopy (amber-gold color, fluid level, handle of malleus position), type B tympanometric curves and absence of otoacoustic emissions. Incomplete medical records or those describing acute otitis media, upper respiratory tract infections on the assessment day or in the last 3 months, neuropathies and craniofacial anomalies were excluded. Data such as gestational age, birth weight, Apgar score, type of feeding and day care attendance were compared between children with and without otitis media with effusion through likelihood tests and multivariate analysis.

**Results:**

25.3% of 184 infants had otitis media with bilateral effusion; 9.2% had unilateral. In infants with otitis media, the following were observed: chronological age of 9.6±1.7 months; gestational age >38 weeks in 43.4% and birth weight >2500g in 48.4%. Otitis media with effusion was associated with winter/fall, artificial feeding, Apgar score <7 and day care attendance. The multivariate analysis showed that artificial feeding is the factor most often associated to otitis media with effusion.

**Conclusions:**

Otitis media with effusion was found in about one third of children younger than 1 year and was mainly associated with artificial feeding.

## Resumo

**Objetivo:**

Determinar prevalência de otite média com efusão em menores de um ano e sua associação com estação do ano, aleitamento artificial, fatores ambientais e perinatais.

**Métodos:**

Estudo retrospectivo com 184 prontuários incluídos de forma randomizada dentre 982 lactentes saudáveis avaliados para testes de triagem auditiva. Diagnóstico de otite média com efusão baseou-se em otoscopia (coloração âmbar-ouro, nível líquido, posição do cabo do martelo), curva timpanométrica tipo B e otoemissões acústicas ausentes. Excluíram-se prontuários incompletos ou que descreviam otite média aguda, infecções de vias aéreas superiores no dia da avaliação ou nos últimos três meses, neuropatias e anomalias craniofaciais. Dados como idade gestacional, peso ao nascimento, Apgar, tipo de aleitamento, frequência à creche foram comparados entre crianças com e sem otites com efusão por meio de testes de verossimilhança e análise multivariada.

**Resultados:**

25,3% dos 184 lactentes apresentavam otite média com efusão bilateral; 9,2% unilateral. Nos lactentes com otite média, observou-se idade cronológica 9,6±1,7 meses; idade gestacional >38 semanas em 43,4% e peso ao nascer >2.500g em 48,4%. Otite média com efusão foi associada ao inverno/outono, aleitamento artificial, Índice de Apgar <7 e atendimento à creche. Já a análise multivariada demonstrou que o aleitamento artificial é o fator mais associado à otite média com efusão.

**Conclusões:**

A otite média com efusão foi encontrada em cerca de 1/3 dos menores de um ano e principalmente associada ao aleitamento artificial.

## Introduction

Otitis media with effusion (OME) is a common chronic condition and usually asymptomatic in children. OME is a risk factor for acute otitis media and for sleep disorders, loss of appetite and ear pain and has psychosocial impacts, which, in the long term, may result in behavioral,^1^ speech and language development disorders.^2^ It is characterized by middle ear inflammation, which is filled with a fluid (effusion) and with no clinical signs of infection.^3^

Its diagnosis in newborns and infants is particularly difficult and inherent to the difficulty of performing the otoscopy in this age group, not only by the size of the ear canal, but also due to the patient's lack of cooperation, the presence of cerumen and the difficulty in removing it.^4^ OME often goes undetected and undiagnosed because it does not have a symptomatic picture as important as acute otitis media. It can spontaneously occur due to reduced function of the eustachian tube or the result of a previous infectious process, among others.^5^

The presence of middle ear secretion and the resulting decreased mobility of the tympanic membrane constitute a barrier to sound conduction and damage the baby's auditory acuity.^6^ Its main sequel is auditory and its main impact is language and cognition impairment.^7^

The difficulties of diagnosing OME during the first year of life make the disease be poorly studied and considering its consequences, it is extremely important to study the factors associated with this age group, as well as to better understand its evolution. Therefore, better therapeutic intervention can be achieved and prevention criteria can be better targeted.

In this context, the aim of this study was to determine the prevalence of otitis media with effusion during the first year of life and its possible association with the season of the year, artificial feeding, perinatal and environmental factors.

## Method

This study was approved by the Institutional Review Board of Hospital das Clínicas da Faculdade de Medicina de São Paulo (1378/09). This is a retrospective study based on the analysis of medical records of infants born at HC-FMUSP.

In 2008 due to technical problems, the neonatal hearing screening was interrupted in our hospital for approximately eight months. A total of 1800 children were not submitted to the tests. They were recalled in 2009 and, of these, 982 children aged 1-12 months came for the assessment.

**Figure 1:**
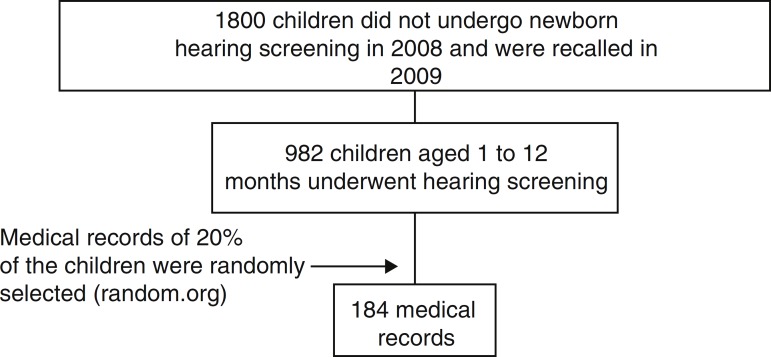
Patient selection.

Records of 20% of the 982 healthy children between one and 12 months, who were recalled for neonatal hearing screening, were randomly selected (random.org) to assess the prevalence of otitis media with effusion. If the selected subject showed any of the exclusion criteria below, the next subject in the random list was included and thus, 184 children's records were selected (Fig. 1).

Children younger than one year that had not been submitted to neonatal hearing screening in the maternity, who were recalled and whose records included detailed data on otoscopy, transient evoked otoacoustic emissions tests and acoustic impedance testing were included. Incomplete records and those belonging to children with acute otitis media and/or upper respiratory tract infection on the assessment day, previous history of acute otitis media in the last three months or severe neurological disease and Apgar<5 at the fifth minute of life and/or craniofacial anomalies (*e.g.*, cleft palate, trisomy 21 and others) were excluded from this study.

The following information had to be included in the medical record for the diagnosis of otitis media with effusion: otoscopy with characteristics of otitis media with effusion and absent response in the transient evoked otoacoustic emissions test and type B tympanometry.^8^ The otoscopy should have three of the following criteria: loss of light reflex, thickening, amber-gold color due to middle ear effusion, air-fluid level, more horizontal appearance of the handle of malleus and retraction pockets. The transient evoked otoacoustic emissions - TOAE - were performed in all children using an Ero-scan device (MAICO^®^, Denmark), comprising the frequency range from 2000-4000 Hz, at the F1 intensity of 65dB NPS - F2 55dB NPS and impedance tests with an Interacoustics AZ7 manual impedance audiometer (1000Hz probe, Interacoustic^®^, Denmark).

The following data on the past clinical history of the children were obtained from the medical records: gender, gestational age, birth weight, Apgar score at five minutes, breastfeeding and daycare attendance. Positive breastfeeding was considered for those children receiving exclusive breastfeeding during the assessment or who were exclusively breastfed for the first six months of life.

The Statistical Package for Social Sciences (SPSS) version 20.0 was used for the statistical analysis. The likelihood test was used to verify the differences in prevalence of otitis media among different seasons of the year and the chi-square test was used to analyze the association of otitis media with other variables.

The multivariate analysis was used to analyze the factors associated with the presence of OME adjusted for confounding variables. Differences for *p*<0.050 were considered statistically significant. For the multivariate logistic regression, the factors that were significantly associated in the univariate analysis were selected.

## Results

The medical records of 184 children were analyzed, with a mean age of 9.6±1.7 months. Table 1 shows the distribution of gender, age, gestational age and birth weight. Forty-five (24.5%) children had otitis media with effusion in both ears and 17 (9.2%) children had it in one ear. A prevalence of otitis with effusion of 33.7% was found. Otitis with effusion was more frequent during the fall and winter (Table 2).

**Table 1:** Gender, gestational age and birth weight distribution.

Category	Frequency	%
*Gender*		
Female	91	49.2
Male	94	50.8

*Age*		
≤6 months	8	4.3
>6 months	176	95.7

*Gestational age*		
>38 weeks	78	42.4
27-34 weeks	34	18.5
34-38 weeks	72	39.1

*Birth weight*		
<1500g	20	10.9
>2500g	89	48.4
1500-2500g	75	40.8

**Table 2:** Prevalence of otitis media with otitis with effusion and season of the year.

Season	Diagnosis		Total
	Normal *n* (%)	OME *n* (%)	*n* (%)
Winter	44 (57.9%)	32 (42.1%)	76 (100%)
Fall	43 (66.2)	22 (33.8)	65 (100%)
Spring	25 (89.3%)	3 (10.7%)	28 (100%)
Summer	11 (73.3%)	4 (26.7%)	15 (100%)
Total	123 (66.9%)	61 (33.2%)	185 (100%)

Likelihood test, *p*=0.016.

Table 3 shows the association of effusion in the middle ear with the male gender; Apgar score at five minutes <7, artificial feeding and daycare attendance. Age, gestational age, birth weight and parental smoking were not associated with OME. However, when applying multivariate logistic regression (Table 4), it can be observed that the most relevant factor associated with otitis media with effusion is the type of breastfeeding.

**Table 3:** Middle ear effusion and association with perinatal and environmental factors.

	Diagnosis				
		Normal		OME	*p*-value
	*n*	%	*n*	%	
*Age*					0.560
≤6 months	5	62.50	3	37.50	
>6 months	117	66.47	59	33.53	
					
*Gender*					0.003
Female	69	56.50	21	33.90	
Male	53	43.50	41	66.10	
					
*Birth weight*					0.069
<1500g	9	7.30	11	18.00	
>2500g	64	52.00	25	41.00	
1500-2500g	50	40.70	25	41.00	
					
*Gestational age*					0.307
>38 weeks	55	44.70	23	37.70	
27-34 weeks	19	15.40	15	24.60	
34-38 weeks	49	39.80	23	37.70	
					
*Type of feeding*					<0.001
Artificial in decubitus	26	21.10	40	65.60	
Artificial sitting	41	33.30	16	26.20	
Maternal>6 months	34	27.60	3	4.90	
Mixed	22	17.90	2	3.30	
					
*Apgar 5 minutes*					0.003
<7	8	6.50	13	21.30	
>7	115	93.50	48	78.70	
					
*Attends daycare* a					0.009
Yes	72	87.80	34	69.40	
No	10	12.20	15	30.60	
					
*Smoker parents* a					0.564
Yes	76	92.70	44	89.80	
No	6	7.30	5	10.20	

a Not all files had data on daycare center attendance and smoker/nonsmoker parents.

**Table 4:** Multivariate analysis of perinatal and environmental factors related to otitis media with effusion in children younger than 1 year.

	Coefficient of regression	Standard error	Odds Ratio	95% confidence interval	*p*-value
Artificial feeding	-0.89	0.21	0.40	0.26-0.62	<0.001
Apgar	-0.92	0.63	0.39	0.11-1.39	0.149
Daycare center	0.91	0.55	2.49	0.83-7.44	0.102

## Discussion

Otitis media with effusion is the most common cause of hearing loss in childhood; it occurs more often during the period of language development and can affect it.^1^

There have been many studies on OME; however, they mostly omit infants or newborns. This condition is a common cause of false positive result in the newborn hearing screening test.^9^

In this series of children younger than one year, OME was found in approximately a third of the children, similarly to what was found by Marchant et al.^10^ However, in their series, 70% of cases of otitis with effusion occurred after recurrent episodes of acute infection.^11^ We excluded data from children with acute otitis media (AOM) in the previous three months because it is common for fluid to persist in the middle ear after this episode. Rosenfeld et al.^2^ described a resolution rate of 50% within one month, 60% within three months and 75% at six months. The presence of middle ear effusion for more than three months characterizes a chronic condition and is of great importance for the development of hearing loss.^6^

OME is a multifactorial disease determined by environmental, socioeconomic and genetic factors.^12^

There was no association of age with OME, probably due to the small sample size. The high number of children with low birth weight and gestational age less than 38 weeks is explained by our hospital profile. It is a tertiary referral center for mothers with high-risk pregnancies.

We found a statistical association for cases of OME diagnosed during fall and winter, which coincides with the months of higher incidence of upper airway infections. In addition to the low temperature, it is noteworthy the fact that São Paulo is a polluted city, with worsening air quality in these months. Brauer et al.,^13^ in a study of automotive traffic pollutants and OME, found a small but significant association between pollutant levels and OME, a fact of potential importance to public health.

Once again it was demonstrated that artificial feeding is a predisposing factor for otitis media with effusion and it is the most important factor among other statistically significant ones. Children who were breastfed for more than six months are less prone to OME, mainly those who do not attend daycare centers.^14^ Breastfeeding during the first year of life has a protective effect for the development of OME, which corroborates the majority of researchers.^15^ This fact is probably related to the positioning of the head, exposure to different microorganisms, improved nutrition and antibacterial or immunological benefits of breast milk.^15^ The supine position without elevation of the trunk was also associated with OME and is consistent with other authors.^16^

Children who attended daycare showed an increased prevalence of OME; these children are exposed to a diversity of viral and bacterial pathogens, in addition to increased person-to-person contact.^17^

We found no association between OME and parental smoking, which differs from other studies that indicate that this factor can increase two-fold the risk of developing otitis media. It should be noted, however, that this sample included a high number of parents who smoked, in both groups.^18^

There was also no association with preterm delivery and low birth weight; however, a lower Apgar score at five minutes was associated with OME. Children with low Apgar scores have other comorbidities not studied in this work and may have been deprived of maternal breastfeeding,^19^ even though we excluded children with very low Apgar scores.

This study extends the current knowledge on the prevalence of OME in children younger than one year; however, it has limitations, such as the retrospective analysis and the absence of data on hearing thresholds, requiring sophisticated tests that are often performed under sedation, which are not carried out in all children at the hearing screening protocol. The external validity of the study is also limited, because of the total number of 1800 children recalled to undergo the neonatal screening, only 982 were able to undergo all the tests.

This study demonstrates once again the importance of breastfeeding as a protective factor for otitis with effusion and also the importance of routinely performing an otoscopy in infants, despite the difficulty to do it, even in asymptomatic children. This practice would influence the therapeutic management of OME, including the referral of these children to the specialist, in order to quickly restore hearing, so important for speech and language development. The hearing loss in OME, although mild, can also lead to behavioral problems such as hyperactivity and inattention, school deficits and cognitive difficulties later in the child's life.^1^

It can be concluded that middle ear effusion was present in approximately one third of infants younger than one year and the factor most strongly associated with the presence of otitis media with effusion was artificial feeding.
